# Understanding activity and physiology at scale: The Apple Heart & Movement Study

**DOI:** 10.1038/s41746-024-01187-5

**Published:** 2024-09-10

**Authors:** James Truslow, Angela Spillane, Huiming Lin, Katherine Cyr, Adeeti Ullal, Edith Arnold, Ron Huang, Laura Rhodes, Jennifer Block, Jamie Stark, James Kretlow, Alexis L. Beatty, Andreas Werdich, Deepali Bankar, Matt Bianchi, Ian Shapiro, Jaime Villalpando, Sharon Ravindran, Irida Mance, Adam Phillips, John Earl, Rahul C. Deo, Sumbul A. Desai, Calum A. MacRae

**Affiliations:** 1 Brigham and Women’s Hospital, Boston, MA USA; 2 Apple Inc, Cupertino, CA USA; 3 University of California, San Francisco, CA USA; 4 Brigham and Women’s Hospital and Harvard Medical School, Boston, MA USA

**Keywords:** Risk factors, Cardiovascular biology

## Abstract

Physical activity or structured exercise is beneficial in a wide range of circumstances. Nevertheless, individual-level data on differential responses to various types of activity are not yet sufficient in scale, duration or level of annotation to understand the mechanisms of discrete outcomes nor to support personalized recommendations. The Apple Heart & Movement Study was designed to passively collect the dense physiologic data accessible on Apple Watch and iPhone from a large real-world cohort distributed across the US in order to address these knowledge gaps.

Longstanding associations of exercise with lower incident disease rates for many disorders have been replicated in large studies with research-grade, as well as consumer-grade, wearables^1–3^. Differential individual responses to exercise are emerging as potential predictors of clinical outcomes in many disorders including diabetes, hip fractures, cancer and rates of cognitive decline^4–7^.

Tailored medical advice on activity remains variable, largely as a result of the limited scope of interventional studies to date, and as a consequence of the wide range of exercise capacity and the heterogeneity of responses to comparable activities^1^. Evidence suggests that most individuals do not meet population recommendations for activity, and it has been proposed that more individualized recommendations might be more effective^8^. Better understanding of the relationships between specific activities and individual physiologic adaptation requires granular documentation of the attributes of different activities and their effects across a range of individuals with discrete response outcomes^8–10^. The convergence of wearable technologies, electronic health records, and modern data science makes such studies feasible.

Apple Watch (Watch) is a multi-sensor wearable which combines passively tracked physiologic metrics (e.g. activity, gait, and heart rate metrics) and incorporates ‘at the wrist’ annotation of events through user-input such as logging workout types. The Apple Heart & Movement Study (AH&MS) was designed to enable longitudinal collection of sensor, activity and health data from individuals to explore the relationships between activity, wellness and health. The study makes possible the principled incorporation of complex physiological models established through deeper phenotyping (such as event follow-up) via Institutional Review Board (IRB)-approved, direct participant outreach.

The current manuscript describes the study design and baseline data from individuals who provided informed consent to participate. The study is ongoing, and data collection continues to evolve with the addition of new sensors, new questionnaires, and other data. Research app, the mobile application participants use to enroll and interact with the study, enables frequent modifications to the study (with IRB approval) facilitating adaptation to new circumstances, such as a pandemic or new data types. Data are time stamped and versioned (both hardware and software) so specific analyses can be framed within the relevant context and App changes can be controlled for. We highlight the core features of the study noting the utility of this approach to incorporate and complement more traditional study frameworks in health and wellness research.

Full methods and summary data from participants followed for at least the initial year are available online. The cohort in the current manuscript was observed until 2021-11-13, two years after the study launch, so that each participant has been observed for at least one full year, and no more than two years. After applying all selection criteria, the initial cohort consisted of 82,809 participants. The detailed characteristics of the participants are reported in Table 1 and Supplementary Table 6. The study cohort is 72% White, 74% male at birth, 74% self-identified as male with a mean age at enrollment is 39.3 years (± 13.1 years). 80% of participants are part-time or full-time employed, 62% college-graduate, 52% married. Current smokers make up 5.3% of the cohort. Mean Body Mass Index (BMI) is 28.4 kg/m^2^ (±6.5 kg/m^2^). The most common prevalent diseases reported by participants were allergies (26.0%), depression (26.0%), and anxiety disorders (24.1%) but despite the age of the study participants, other medical conditions are reported at notable rates (Table 2) with 61% of participants currently taking at least one medication (Supplementary Table 7).

**Table 1:** Participant characteristics, according to their earliest submitted self-reports

Characteristic	Value	Characteristic	Value
Age (yrs), median [IQR]	37.2 [29.4, 47.3]	Employment	–
Race	—	Employed for pay, % (*N*)	80.5 (66,637)
White, % (*N*)	71.8 (59,421)	In school, % (*N*)	6.1 (5085)
Hispanic, % (*N*)	11.3 (9346)	In retirement, % (*N*)	5.3 (4418)
Asian, % (*N*)	6.6 (5424)	Unemployed, % (*N*)	3.6 (3018)
Black, % (*N*)	4.3 (3544)	Unable to work, % (*N*)	2.0 (1638)
> 1 race, % (*N*)	3.2 (2656)	Taking care of house/family, % (*N*)	1.7 (1391)
Other, % (*N*)	1.1 (914)	Prefer not to answer, % (*N*)	0.7 (571)
None of these, % (*N*)	1.3 (1094)	Missing, % (*N*)	<0.1 (51)
Prefer not to answer, % (*N*)	0.5 (401)	Marital Status	–
Missing, % (*N*)	<0.1 (9)	Married, % (*N*)	52.3 (43,270)
Gender Identity	–	Never married, % (*N*)	27.6 (22,860)
Man, % (*N*)	73.6 (60,932)	Member of unmarried couple, % (*N*)	10.1 (8373)
Woman, % (*N*)	24.7 (20,452)	Divorced, % (*N*)	7.2 (5944)
Gender queer/nonbinary, % (*N*)	0.6 (477)	Separated, % (*N*)	1.2 (954)
Multiple identities, % (*N*)	0.4 (337)	Widowed, % (*N*)	0.8 (635)
Prefer not to answer, % (*N*)	0.3 (286)	Prefer not to answer, % (*N*)	0.9 (718)
Other, % (*N*)	0.3 (246)	Missing, % (*N*)	< 0.1 (55)
Missing, % (*N*)	< 0.1 (79)	Subjective Social Status (1 = lowest)	–
Sex assigned at birth	–	1 to 4, % (*N*)	15.0 (12,414)
Male, % (*N*)	74.4 (61,612)	5 to 6, % (*N*)	36.7 (30,424)
Female, % (*N*)	25.2 (20,896)	7 to 10, % (*N*)	48.0 (39,777)
Intersex, % (*N*)	< 0.1 (56)	Missing, % (*N*)	0.2 (194)
Prefer not to answer, % (*N*)	0.2 (167)	Place of residence	–
Missing, % (*N*)	< 0.1 (78)	South, % (*N*)	34.6 (28,653)
Intersex	–	West, % (*N*)	29.3 (24,280)
No, % (*N*)	97.0 (80,361)	Midwest, % (*N*)	18.7 (15,487)
Yes, % (*N*)	0.9 (742)	Northeast, % (*N*)	17.2 (14,222)
Prefer not to answer, % (*N*)	2.0 (1627)	Territories, % (*N*)	0.2 (158)
Missing, % (*N*)	< 0.1 (79)	Missing, % (*N*)	< 0.1 (9)
Education	–	BMI (kg/m^2^), mean ± SD	28.4 ± 6.5
College and above, % (*N*)	61.7 (51,134)	Current smoker, % (*N*)	5.3 (4372)
Some college, % (*N*)	27.5 (22,753)		
High-school graduate, % (*N*)	9.2 (7593)		
Less than high school, % (*N*)	1.0 (853)		
Prefer not to answer, % (*N*)	0.5 (426)		
Missing, % (*N*)	< 0.1 (50)		

Characteristics are reported as either: 1) mean value ± standard deviation; or, 2) the percentage of the 82,809-person cohort that identifies with the characteristic, along with the associated absolute number of participants; or, 3) median value, with 1^st^ and 3^rd^ quartile.

**Table 2:** Past and current medical conditions, derived from each participant’s earliest submitted Medical History survey

Condition	% (N)
Allergy	26.0 (21,526)
Depression	26.0 (21,497)
Anxiety	24.1 (19,963)
High cholesterol	20.0 (16,583)
High blood pressure	18.6 (15,400)
Vision loss	17.7 (14,617)
Asthma	15.7 (12,993)
Arthritis	11.8 (9798)
Sleep apnea	11.5 (9509)
Chronic back disorder	11.3 (9340)
Hearing loss	10.7 (8886)
Arrhythmia (not A-fib)	5.8 (4819)
Chronic neck disorder	5.1 (4223)
Diabetes	4.8 (4008)
Thyroid disease	4.7 (3889)
Cancer	3.3 (2717)
Kidney disease	3.1 (2,570)
COPD	2.4 (1993)
Atrial fibrillation	2.1 (1740)
Urinary incontinence	1.9 (1589)
CAD	1.7 (1367)
Osteoporosis	1.6 (1307)
Hip or knee replacement	1.5 (1242)
Neuropathy	1.3 (1089)
Heart attack	1.1 (932)
Stroke or TIA	0.7 (597)
Heart failure	0.6 (478)
Peripheral artery disease	0.5 (403)
Pacemaker	0.4 (321)
Liver disease	0.3 (255)
Pregnant	0.2 (145)
No survey submitted	19.8 (16,413)

Table shows the percentage of the 82,809-person cohort that reports each condition with the associated absolute number of participants in parentheses.

To demonstrate the range of the HealthKit data shared by the cohort during a single week, we aggregated results over a representative 7-day period to average out weekly cycles in participant activity (Table 3). The most common activity type was walking, which was shared at least once by 20.0% of the cohort. A total of 25,304 (30.6%) people in the cohort shared at least one workout during the week of observation, averaging, among them, 6.54 workouts per person.

**Table 3:** The 27 workout types shared by at least 100 participants during final 7 days of observation period

Activity Type	Participants, % (*N*)	Workouts/participant
Walking	20.0 (16,566)	3.8
Cycling	6.5 (5416)	3.6
Running	6.4 (5326)	2.8
Traditional strength training	4.7 (3867)	2.7
Functional strength training	3.7 (3037)	2.7
Other	3.2 (2652)	3.8
Yoga	2.9 (2374)	2.5
High-intensity interval training	2.8 (2291)	2.6
Elliptical	1.8 (1470)	3.1
Core training	1.3 (1078)	2.0
Hiking	1.2 (963)	1.8
Cooldown	1.0 (862)	2.2
Rowing	0.9 (742)	2.5
Cross training	0.7 (616)	2.7
Preparation and recovery	0.7 (592)	2.7
Swimming	0.6 (516)	2.3
Mixed cardio	0.6 (506)	3.3
Flexibility	0.6 (461)	2.9
Pilates	0.5 (451)	1.7
Stair climbing	0.5 (378)	2.0
Mind and body	0.4 (369)	3.7
Cardio dance	0.4 (369)	1.8
Golf	0.3 (268)	2.0
Tennis	0.3 (258)	2.1
Basketball	0.1 (120)	1.7
Climbing	0.1 (118)	1.8
Soccer	0.1 (103)	1.5

*Activity Type*: the label used within HealthKit for a given type of workout. *Participants*: The number of individuals, *N*, who shared at least one such workout also presented as a percentage of 82,809-person cohort. *Workouts per participant*: Total number of workouts of that type, divided by *N* participants who shared that type of workout.

Workouts are a special case of exercise tracking, within the general class of HealthKit samples, many other types of which exist (Supplementary Table 8). Among these, the sample types that are most commonly shared are those generated by everyday Apple Watch-wear and passively collected by sensors and software native to Watch. Accordingly, step count, heart rate and stand hours are shared with the study during the specific week represented in about half of the cohort. Less commonly shared sample types include: ‘Mindfulness’ sessions (shared by 5.5% during the specific week), which record a mindful session that is guided by Watch but requires active participant engagement; and high heart-rate event (shared by 2.4% during the specific week), which is passively collected by Watch but which is not a frequent event for healthy participants. Other data supplied by connected third party sensors are less frequently shared. For comparison, participant confirmed workouts are included, when initiated by a participant or confirmed from an auto-detected workout.

The cohort included 66,752 people (80.6%) who, for at least one day in their initial year post-enrollment, had an Apple Watch capable of recording an ECG paired with Research app. A single-lead ECG can be recorded at any time through the ECG app by holding the watch crown for 30 seconds. Within this subset, there were 55,740 people (83.5% of those wearing an ECG-capable Watch) who recorded and shared an ECG in their first-year post-enrollment, for a total of 1,132,473 ECGs (see Table 4). 25,402 ECGs (2.2%) were classified as showing atrial fibrillation, representing 1641 participants (2.0% of the cohort (see Supplementary Table 9). Of these participants, 477 (29.1%) had reported that they were known to have atrial fibrillation, suggesting that symptoms were often a driver for the specific recordings.

**Table 4:** ECG summary table for Year 1

Watch Model - Cohort Description	
Number of participants in cohort	82,809
Number of participants without ECG-capable Watch (Series 1–3 or SE)	16,051
Number of participants with ECG-capable Watch (Series 4, 5, 6)	66,752
Number of participants with unknown Watch series	6
**ECG data shared**	
Number of participants with at least one ECG in first year post-enrollment, *N* (% with ECG-capable Watch)	55,740 (83.5)
Total ECGs taken within first year	1,132,473
Median number of ECGs, among participants with at least one ECG in the first year	8

The Apple Health app allows users to download clinical health records from participating institutions by signing into their healthcare provider’s portal and choosing to share Fast Healthcare Interoperability Resources (FHIR) data with HealthKit. To date, the proportion of participants who have been able to share these data types is modest (~10%) as a consequence of local FHIR compliance and the process required. In the current cohort, 8408 people shared at least one such record with our study.

To measure participant engagement with the study over time, we present two very basic indices which complement the more detailed reports of survey data and HealthKit sharing online. These are: 1) how often a participant’s Apple Watch shares the HealthKit sample type “Stand Hour” with Research app; 2) how often a participant’s Research app uploads any kind of data to Apple’s secure study servers. Figure 1 shows these two indices of participation on discrete time scales. Panel a shows the fraction of the cohort who did not participate on any given day post-enrollment (00:00:00 to 23:59:59 UTC). Panel b shows the fraction that never participates at *any time after* a given day post-enrollment and can be regarded as a measure of the incidence of cohort dropout over time, specifically a measure of the fraction that becomes indefinitely inactive, according to that index of participation. Thus, for both of these indices, the decline in daily participation over time is largely attributable to the accumulation of permanently inactive participants over time, and less a consequence of degradation of study engagement among active participants. For example, 44% of the cohort does not share a Stand hour sample on day 365 post-enrollment, but this is not much larger than the 34% of the cohort that has already stopped sharing Stand hour samples at all times after day 365.

**Fig. 1:**
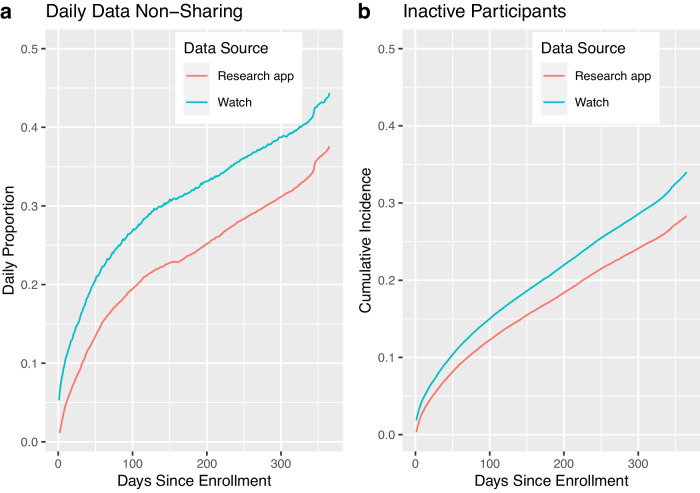
Four measures of participation vs. time since enrollment. Blue lines display the proportion of participants who have not contributed a Stand Hour sample, measured by their Watch. Red lines display the proportion of participants who have not uploaded any data at all from their Research app. Panel (**a**) shows the fraction of the cohort that do not participate in these two ways on a given day, post-enrollment. Panel (**b**) shows the fraction that no longer participates at any time after a given day, post-enrollment as a measure of the cumulative incidence of dropout from the study.

AH&MS also includes a series of 16 surveys sent to participants and outlined in Table 5. Except for the 5 surveys that are triggered by rare events detected by Watch, almost all the surveys have a participation rate greater than 70%. Figure 2 shows the response rates vs. time since enrollment for two surveys delivered with the highest frequency - the monthly Stress Scale survey and the quarterly Changes in Health survey. A decreasing trend of response rate over time is clearly visible in Fig. 2, starting at 69.55% and gradually dropping to 32.48% after one year. As expected, due to the burden of survey completion on participants, this one-year decline is larger in both absolute and relative terms than the decline in active users as measured by Research app uploads in Fig. 1 Panel b, which shows only 28% of the cohort becoming indefinitely inactive at one year. A similar decreasing trend of response rate over time is observed in the Changes in Health survey.

**Table 5:** Number of participants in the cohort who complete at least one survey in their first year post-enrollment

Survey		Number of participants with >= 1 submission, % (*N*)	Month delivered, post-enrollment
SCHEDULED	Demographics	100.0 (82,809)	0
	Risk of Falling	85.2 (70,570)	1
	Mobility Status	80.8 (66,877)	2
	Stress Scale	80.4 (66,562)	3
	Mental Health	80.0 (66,239)	2
	Medical History	78.9 (65,369)	1
	Physical Activity	78.0 (64,558)	2
	Health Behaviors	75.1 (62,214)	1
	Medications	75.1 (62,198)	1
	Disability Assessment	72.8 (60,318)	3
	Changes in Health	67.9 (56,227)	4
TRIGGERED	Potential Fall (low prob.)	32.6 (26,987)	NA
	Potential Fall (high prob.)	8.8 (7312)	NA
	ECG Follow-Up	0.9 (762)	NA
	Irregular Rhythm Follow-Up	0.8 (629)	NA
	Take an ECG	0.6 (480)	NA

Percentage is calculated among the entire 82,809-person cohort.

**Fig. 2:**
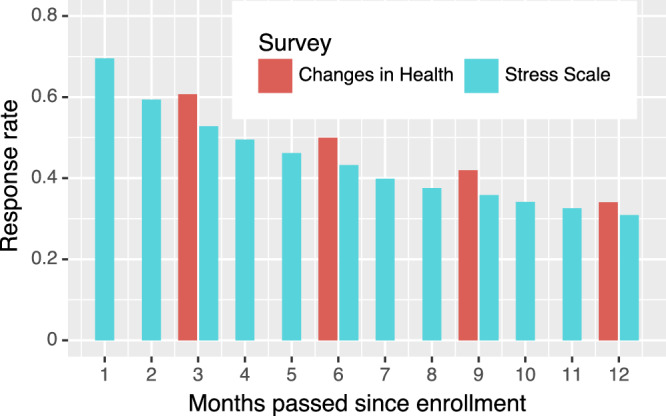
Response proportions for the monthly Stress Scale survey and the quarterly Changes in Health survey over time since enrollment. For the Stress Scale survey, percentage is calculated among 42,181-person sub-cohort since only people enrolled after 2020-05-01 are considered to avoid any frequency change in survey delivery. For Changes in Health survey, the whole 82,809-person cohort is considered.

AH&MS is similar in scale to several other studies of wearable data and has already demonstrated the potential to overcome many of the constraints in prior studies of activity and physiologic adaptation^1^. The combination of multiple independent sensors, granular ‘at the wrist’ annotation of physiologic data, and the access to health conditions and health outcomes within Apple’s ecosystem is distinctive and brings high dimensionality to a large cohort without discrete medical indications. AH&MS enables the longitudinal investigation of a broad range of validated physical performance attributes combined with dense contextual metadata and extensive outcome metrics. The sustained survey engagement of participants is considerably improved from prior studies of serial health questionnaires. Among the features which are likely to prove of greatest utility are dynamic recruitment strategies, the passive collection over time and trending of validated reference biometrics (such as VO_2_max, heart rate recovery, etc), a rapidly modifiable study App^11,12^, the availability of participant generated high-resolution annotation, and consented access to clinical health record and claims data.

We anticipate that the AH&MS study design will directly address the need to understand relationships between longitudinal real-world wearable trajectories and the cross-sectional clinical or biological research data collected in typical biomedical research studies. Many existing epidemiologic cohorts lack longitudinal objective data on core lifestyle characteristics, while collecting rigorous outcome data for specific causes of mortality and morbidity. AH&MS collects and trends extensive behavioral and activity data elements, in addition to expanded demographic, anthropometric and general lifestyle data elements from HealthKit. Combining these datatypes with research sensor and usage data allows for unique exposure and outcomes assessments.

AH&MS has been designed to allow direct comparisons with traditional epidemiologic studies and randomized clinical trials through shared minimal common datasets. The ability to contact individual participants also enables deep phenotyping based on structured sampling. Longitudinal trends in complex physiologic metrics can be compared to interpolated external measurements, which in the past have been measured in cross-sectional fashion in highly selected populations. Models trained on sub-cohorts that have been deeply phenotyped can then be deployed across the entire AH&MS or in any traditional clinical study format.

The availability with Apple Watch of real-time ‘at the wrist’ annotation of activities and associated passive recording offers a granular ground truth to AH&MS. Research app is highly configurable to enable rapid modification and to accommodate emerging or secondary research questions such as the study response to the SARS Co-V-2 pandemic.

AH&MS also has ongoing access to consented data from participant clinical health records and data from “Blue Button” surfacing of records through FHIR application programming interfaces (APIs), and both these data types are accumulating after initial delays due to low healthcare utilization during COVID. As the process of incorporating EHR data into AH&MS is simplified, we are also exploring approaches to contemporaneous validation of specific incident diagnoses for the study. These datasets will allow the integration of long-term wearable trajectories with both prevalent and incident healthcare data, laying the foundation for continuous or semi-continuous data trajectories from wellness to disease.

The study has several limitations which must be considered. The study is limited to Apple iPhone and Apple Watch users, and there are limits regarding generalizability to other populations. Naturalistic study design, in both sampling and in analysis, may introduce discrete confounding as may the ongoing addition of new algorithms or new sensors. The loss to follow-up estimated from monthly survey response rates is higher than other retention metrics, though the survey characteristics and delivery cadences have not yet been optimized for response or retention rates. Other forms of missing data are prevalent, and though Watch wear information can assist in interpretation, this missingness must be accounted for in any analyses. These challenges include timing of data loss with respect to phone upgrade, the characteristics of participation prior to study drop-out, frequency of contact, survey length, prior survey completion and many others. The patterns of dropout in the initial year of the study skew participation further in the direction of initial recruitment biases, emphasizing the need for systematic approaches to drive representativeness in participant recruitment and retention. AH&MS extends age, gender, racial and ethnic diversity, but remains incompletely representative and we have added quantitative strategies to both recruit and retain relevant populations. These include local and social media campaigns and friend or family member recruitment. Female study recruitment has steadily grown over time and retention of this demographic is high. We anticipate much more granular understanding of study dropout mechanisms and their prevention as the study progresses.

Understanding physiologic responses to external challenges offers systems-level information on the physiologic set points of the individual and has been shown to lead to much more rigorous discrimination of intrinsic differences between individuals than passive cross-sectional measurement. These details highlight the potential to enable much more specific recommendations on optimal activity patterns for the individual user. The current study has been designed to lay the foundation for a broader and deeper interrogation of health and fitness metrics and to relate these parameters to outcomes in health and fitness.

## Methods

### Study design

This is a mobile application-based longitudinal cohort study involving the collection of sensor, survey, and health data. Participants were informed about the study through IRB approved materials, including study websites managed by Apple, the American Heart Association (AHA) and Brigham and Women’s Hospital. Participants are asked to complete a series of surveys and to consent to sharing data collected from their Health app (which uses the HealthKit framework) and sensor data obtained directly from iPhone and Apple Watch (Supplementary Table 1). The Research app framework enables participants to opt into and out of sharing specific types of health data with the study. The study began enrolling on November 14, 2019 with a goal of enrolling up to 500,000 participants. The planned duration of this study is 5 years, that is until November 2024, with a potential for extension or additional long-term follow-up. The study was approved by the Advarra Central Institutional Review Board (PRO00036784) and registered to ClinicalTrials.gov (ClinicalTrials.gov Identifier: NCT04198194). There is no compensation for participation.

Survey questions were designed to enable comparison with data from US health studies of similar scale, such as National Health and Nutrition Examination Survey (NHANES) developed by the Centers for Disease Control and Prevention^13^ and the All of Us Study, a large research program sponsored by the National Institutes of Health^14^. Questions were modified to support delivery within the mobile app user interface and were standardized, where relevant, across all three of the simultaneously launched Apple studies in 2019, including the Apple Women’s Health Study^11^ and the Apple Hearing Study^12^.

The Research app user interface was designed to be simple and intuitive while enabling data collection, through tasks such as survey completion, to be distributed over time, so as to reduce participant burden. The estimated time demand for survey responses was approximately 30 min in the first month, with 10 min per month during ongoing participation as additional surveys are triggered based on responses or the need for additional data collection (such as data on COVID exposures).

This study was designed with participant privacy in mind. Participant data are coded and encrypted while in transit and at rest. Coded data are stored in a system designed to meet the technical safeguard requirements of the Health Insurance Portability and Accountability Act (HIPAA). To maximize participant privacy and the confidentiality of health data and to minimize the risk of unauthorized access to participant data, a discrete workflow was created which enables Apple access to coded study data while restricting access to identifiable information such as name and contact information to a limited number of authorized staff at BWH. This access also allows contact between BWH and study participants through a discrete workflow which is inaccessible to Apple.

### Eligibility, screening, and consent

Similar to the Apple Women’s Health Study and Apple Hearing Study^11,12^, eligibility criteria include access to an iPhone with Research app installed, comfort communicating in written and spoken English, residence in the United States, aged at least 18 years old (at least 19 years old in Alabama and Nebraska, at least 21 years old in Puerto Rico), unique use of iCloud account or iPhone, and willingness to provide informed consent to participate in the study. An additional requirement for AH&MS includes use of an Apple Watch (Series 1 or later) paired with an iPhone at the time of enrollment.

Prior to enrollment, participants complete a profile which includes information such as name, date of birth, email, phone number, current region and state of residence. These data are used to confirm eligibility. For AH&MS, Research app is also able to confirm if an Apple Watch is paired to the iPhone. If the requirements for age, location, and Watch pairing status are met, individuals are able to continue to study onboarding, including reading and signing the informed consent form (ICF), HIPAA Authorization, and California Bill of Rights (if applicable). Since study launch, there have been revisions to survey frequency and to questions within the surveys, introduction of new surveys, and updates to the ICF.

Once onboarding is completed, newly enrolled participants are immediately allocated two “tasks”, specifically, the demographic survey along with a brief guide “Using your Apple Watch to Contribute” explaining that both logging workouts on Watch and taking ECGs (for those with Watch Series 4 or higher) are valuable to the study.

### Ongoing recruitment

With institutional review board (IRB) approval, a study website hosted by Brigham and Women’s Hospital/Harvard Medical School was also made public. The American Heart Association (AHA) simultaneously launched an informational website to spread awareness of the study (November 2019), including IRB-approved social media and email campaigns (October 2020). The AHA also launched a website on heart.org to raise awareness of the study and broaden ongoing recruitment efforts. In addition, a new Research app feature was introduced and launched in October 2020 to allow updates to be sent directly to participants in the app to encourage continued participation, maximize participant engagement, provide study insights, and increase recruitment efforts.

### Survey data—at enrollment and annually thereafter

On enrollment, participants in AH&MS responded to the Research Profile Survey within the Research app and to the Demographics Survey. The data included the year of birth, state of residence, race and ethnicity, marital status, employment status, education level, gender identity, sex assigned at birth, and subjective social status. Within the first month after enrollment participants received the following surveys: a Risk of Falling survey (based on STEADI survey, first 12 questions)^15^, a Medical History survey, a Medications survey, and a Health Behaviors survey (based on the Alcohol Use Disorders Identification Test (AUDIT-C)^16^ and All of Us study, NIH)^14^ delivered through Research app. The Activity Status survey, a questionnaire related to physical activity, was administered at the beginning of month two. Annual surveys are delivered on a staggered timeline to reduce participant burden and anticipated to take approximately 5 min each. Each survey expires approximately 28 days after delivery, except the Demographics survey, which never expires.

### Scheduled interim surveys and timing

The participants also received quarterly surveys, specifically, the Mental Health survey (PHQ-2, GAD-2)^17^, the Activity Status survey (Modified Rosow-Breslau)^18^, the Perceived Stress Scale survey (PSS-4)^19^, the Disability Assessment survey (WHODAS 2.0)^20^, and reported outcomes related to changes in health in the Changes in Health survey. These surveys are also staggered across months two, three, and four, with an estimation of 10 min for survey completion each month.

### Triggered surveys and timing

The study design also includes surveys sent to participants only if they meet certain criteria occurring during the period they are enrolled in the study. There were 3 triggered surveys at study launch related to sensor data observed on Apple Watch, with an estimated 5 min required to complete each survey.

The Irregular Rhythm Follow-up survey is administered 3 months after a participant receives an irregular heart rhythm notification while wearing Watch versions that support this functionality, which Watch can detect passively. The Irregular Rhythm Follow-up survey is designed to understand what participants did in response to the notification and is limited to a single administration every 90 days if the trigger criterion is met.

The second triggered survey, called ECG Follow-up, is administered 3 months after a participant receives an atrial fibrillation result during use of the Apple Watch ECG feature. The questions are related to any actions taken and care received after the result is given to the participant. This is designed to only be administered once to each participant whose ECG exhibits evidence of atrial fibrillation.

The Potential Fall survey, which is triggered when Apple Watch detects motion signatures that suggest the participant has experienced an impact, such as a hard fall. This questionnaire is delivered the day after the fall event is detected by the Research app and is designed to verify that the event was a fall, capture the participant’s activities during the fall, and assess if there were resulting injuries. To reduce the burden for participants who fall frequently, the study limits the number of Potential Fall surveys to 4, for events classified as falls with high probability, and limits delivery to 1 survey per month for events classified as falls with low probability.

### Participant-approved follow-up to triggered surveys by study staff

A workflow was implemented to collect more detailed information from participants who agree to be contacted regarding events, such as a potential fall, that meet protocol-defined criteria. To contact participants, BWH staff use a secure mobile application and workflow to access contact information including name, phone, and email that is not accessible by Apple. Using an IRB- approved script, answers to detailed questions regarding the event are logged in a formatted structure, that is reviewed for accuracy and removal of any personally identifiable data and then aggregated with sensor and survey data.

### Research sensor and usage data

Participants consented to the collection of a set of data (approved by the participant) of derived metrics retrieved from iPhone sensors and a paired Apple Watch. These data included ECG details, heart rate via the optical sensor (PPG), elevation (barometric pressure), motion (accelerometer, gyroscope), speed and distance (derived from GPS) and other sensor data such as the “on-wrist state,” pedometer data, and fall statistics summarized in Supplementary Table 2. Once enrolled, participants can opt into or out of sharing specific types of data with the Study using controls accessible within Research app.

### HealthKit data

Using the HealthKit framework to collect both passively and manually added data types which participants have consented to share, provides a central repository for health and fitness data on iPhone and Apple Watch. Under such permission, specific apps write and read data using HealthKit which in turn can be accessed and shared with Research app while maintaining participant privacy and control. HealthKit stores data merged from multiple sources and contains data types such as heart rate, work out data, sleep analysis, and clinical health records (lab tests, diagnoses) from clinical interfaces. For this initial descriptive analysis, we have provided sampling from a randomly chosen (and typical) week to demonstrate the extent of the data collected.

### Participation

This study was designed to allow participation in the following complementary ways: (1) response to survey questions in Research app; (2) contribution of HealthKit data; (3) contribution of sensor and usage data, which include sensor-based data streams from SensorKit (SK); and (4) response to direct outreach from study staff if specific IRB-approved criteria are met. In general, a participant is considered to be actively participating when contributing data through any of these methods.

### Defining demographic variables

We used the same questionnaire that was used to determine the race and ethnicity in the NIH-sponsored All of Us Study and we classified responses into traditional reporting of race and ethnicity. All three of the Research app studies used the MacArthur Scale of Subjective Social Status^21^. This scale has been observed to correlate with health status across the lifespan. Notably, the MacArthur Scale is correlated with objective socioeconomic status (SES) but has the benefit of broader applicability as a marker of social status than simple objective measures of SES in non-White populations. We arbitrarily categorized the responses into the following categories: 1 to 4 corresponding to low, 5 to 6 corresponding to middle, and 7 to 10 corresponding to high.

### Planned statistical analyses

Statistical analyses are planned for the following 3 categories of study: (1) longitudinal analyses of survey data, (2) longitudinal analyses of passively collected iPhone and Apple Watch data, and (3) analyses of associations between the survey data and passively collected data including clinical health record data. In each category, we will perform exploratory descriptive analyses and formulate more specific hypothesis-driven models.

For all 3 types of analyses, we will use longitudinal extensions of regression methods, such as linear and generalized linear mixed models, statistical learning techniques for high-dimensional data, and functional data analysis methods. For the longitudinal analysis of the survey data, we will quantify the associations among both participant characteristics and risk factors and specific functional outcomes, and how these associations vary across the age range of the study population. For the longitudinal analysis of passive data, we will perform individual-level analyses to identify the possible change points in behaviors over time and how they relate to subsequent health outcomes. For the longitudinal analysis of passive and survey data, the predictors will initially be the daily summary statistics derived from passively collected data and the outcomes of interest will consist of all the items on which survey data are available. We will train machine learning models on objective outcomes defined within the study itself and use these models to classify specific time to event trajectories.

Participants who enrolled but failed to submit the Demographics survey are excluded from the cohort studied in this article. A Welch two-sample, two-sided t-test was performed to compare mean age at enrollment between included and excluded participants. A Pearson’s chi-squared test with simulated p-values was performed to compare the distribution of geographic regions between included and excluded participants. This analysis was performed using R version 3.6.0 (base R).

## Data

### Baseline Characteristics of Participants

We present characteristics, measured as close to enrollment as possible, for the cohort of study participants who enrolled in the study in its first year, 2019-11-14 through 2020-11-13. The cohort in the current manuscript was observed until 2021-11-13, two years after the study launch, so that each participant has been observed for at least one full year, and no more than two years.

We did not include the following: (1) data used to test Research app for quality assurance purposes (*n* = 29); 2) cases where eligibility became ambiguous after enrollment (e.g. participant modified dates of birth or address after enrollment to imply age less than local age of consent (*n* = 100); or, (3) participant did not complete the Demographic survey after enrollment (*n* = 1751). After applying all selection criteria, the initial cohort as of 2020-11-13 consists of 82,809 participants.

For the characteristics reported in Table 1, the value represented for each participant is the earliest value recorded by Research app for that participant and characteristic. Multiple values may occur when a participant is enrolled for long enough that Research app presents them with a survey for a second or third time, for example a 1^st^ annual Demographics survey and a 2^nd^ annual Demographics survey. Participants are also able to edit, at any time, their date of birth and place of residence in the profile maintained for them by Research app. In cases where a participant edits this information after enrollment, we present only the earliest value that they share. In the event that a participant edits their data, an additional eligibility check is run and the individual may be removed if ineligible (e.g. moves to a new state that has a higher minimum age limit for participation).

Comparison of this 82,809-person cohort to the 1751 participants who did not respond to a Demographic survey is performed in Supplementary Table 3, 4 and 5 on the basis of participants’ age and state of residence at enrollment (these data are obtained by Research app prior to enrollment, regardless of whether a participant submits the Demographics survey). The 1751 excluded participants are 3.2 years younger than the cohort, on average (95% CI = [3.79 y to 2.58 y]).

The study cohort is 72% White, 74% male at birth, 74% self-identified as male (Table 1). Mean age at enrollment is 39.3 years (± 13.1 years). 11% of the cohort is Hispanic; details of the racial makeup of this Hispanic population are in Supplementary Table 6. 80% of participants are part-time or full-time employed, 62% college-graduate, 52% married. Current smokers make up 5.3% of the cohort. Mean BMI is 28.4 kg/m^2^ (±6.5 kg/m^2^).

2,684 participants (3.24% of the cohort) withdrew from the study within one year of enrollment. Among those who withdrew, 25% withdrew in their first 13 days, and 50% withdrew within 111 days. Among those who withdrew < 1% were automatic withdrawals triggered by an update to a participant’s state of residence or date of birth that rendered them no longer eligible to continue in the study. More details about withdrawal rates can be found in Supplementary Fig. 1.

The most common prevalent diseases reported by participants were allergies (26.0%), depression (26.0%), and anxiety disorders (24.1%) (Table 2), but despite the relatively young age of the study participants, they reported many other medical conditions at notable rates.

Among all participants in the cohort, 61% report currently taking at least one medication (Supplementary Table 7). The most commonly reported medications were NSAIDs (27%), antidepressants (20%), and either ACEIs or ARBs (11%).

### Health-data sharing in a single week

To demonstrate the variety of the HealthKit data shared by the cohort during a single week, we aggregated results over a 7-day period to average out weekly cycles in participant activity (data not shown). We chose the final week in our observation period (2021-11-07 through 2021-11-13) in particular since all participants would have been enrolled for at least one year at that point, and since—at two years after study launch—it was the point closest to the middle of the study’s 5-year period. Comparison of this week to 145 other weeks between 2019-11-14 and 2022-09-01 established that the period chosen is representative of a typical week (See Supplementary Fig. 2A and 2B).

Table 3 shows the most common of the 82 types of workout sample logged into HealthKit and shared with the Research app by at least 100 participants. For each activity type, the table gives the number of participants who shared at least one of that sample type in that week and gives the average number of activities of that type per participant (among those who performed that activity). The most common activity type was walking, which was shared at least once by 20.0% of the cohort. A total of 25,304 (30.6%) people in the cohort shared at least one workout during the week of observation, averaging, among them, 6.54 workouts per person.

Workouts are a special case of exercise tracking, within the general class of HealthKit samples, many other types of which are listed in Supplementary Table 8, which presents HealthKit data from the week starting 2021-11-07 with 100 or more participants contributing to each data type. This sample is restricted to year 1 enrollees who were active during this specific week in year 2. Among these, the sample types that are most commonly shared tend to be those that are generated by everyday Watch-wear and that are passively collected by software and sensors native to Watch. Accordingly, step count, heart rate and stand hours are shared with the study during the specific week represented by Supplementary Table 8 by about half of the cohort. Less commonly shared sample types include: ‘Mindfulness’ sessions (shared by 5.5% during the specific week), which record a mindful session that is typically guided by Watch but which requires active participant engagement; and high heart-rate event (shared by 2.4% during the specific week), which is passively collected by Watch but which is not a frequent event for healthy participants. Other data supplied by connected third party sensors, for example blood glucose (shared by 1.1% during the specific week), are less frequently shared.

For comparison, participant confirmed workouts are included, when initiated by a participant or confirmed from an auto-detected workout. This attribute, and the fact that structured physical exercise is not a frequent part of everyday activities, means that our dataset contains many fewer workout samples than, for example, heart rate samples or step count samples.

### ECGs

The cohort included 66,752 people (80.6%) who, for at least one day in their initial year post-enrollment, had an Apple Watch capable of recording an ECG paired with Research app. A single-lead ECG can be recorded at any time through the ECG app by holding the watch crown for 30 s. Within this subset, there were 55,740 people (83.5% of those wearing an ECG-capable Watch) who recorded and shared an ECG in their first-year post-enrollment, for a total of 1,132,473 ECGs (see Table 4). During the defined data collection period for this cohort, there were two tasks that encouraged participants to take an ECG. For all participants with a capable Watch, one task is presented at enrollment that encourages taking an ECG and an additional bi-weekly task was added in April 2021 that continued through January 2022 to take an ECG and record the result via multiple choice response.

25,402 ECGs (2.2%) were classified as showing atrial fibrillation, representing 1641 participants (2.0% of the cohort). Other classifications are shown in Supplementary Tables 9 and 10. This collection period includes both ECG version 1 and ECG version 2, which became available on WatchOS 7.2 and iOS 14.3, originally released in December 2020, with expanded ECG classification capability.

### Clinical health records

The Apple Health app allows users to download clinical health records (FHIR format) from participating institutions by signing into their healthcare provider’s portal and choosing to share FHIR data with HealthKit. Study participants may elect to share this data with our study. To date, the proportion of participants who have been able to share these data types is modest (~10%) as a consequence of local FHIR compliance and the process required. In the cohort, 7757 people shared at least one such record with our study in their initial year post-enrollment.

### Measures of participation over time

To measure participant engagement with the study over time, we present two very basic indices which complement the more detailed reports of survey data and HealthKit sharing above. These are: (1) how often a participant’s Apple Watch shares the HealthKit sample type “Stand Hour” with the Research app; (2) how often a participant’s Research app uploads any kind of data to Apple’s secure study servers.

Each Stand Hour sample is an estimate by Apple Watch of whether or not the participant has stood and moved for at least 1 min during a given hour of the day. If Watch is not on the wrist and powered on, then no sample is created.

We use the presence of Stand Hours as a proxy for Apple Watch wearing, since this parameter is passively collected and because Watch logs an indication of “Stood” or “Idle” each hour the participant is wearing the device. We interpret the absence of Stand Hour samples on a given day to mean that the participant was not wearing Watch that day. We note, however, that at least two other conditions might result in the absence of a Stand Hour sample: (1) the data upload path from Watch to the Research app and then to the AH&MS servers was not active (e.g. lack of connectivity); or (2) the participant has opted out of sharing Stand Hours with the Research app after enrollment, a user setting which it is not possible to directly ascertain but which our data suggest is unusual for current study participants.

Our second definition of participation is based on a more modest requirement: that a participant’s Research app has uploaded *any data* to the study servers on any given day. Such an upload might represent a Stand Hour sample or other health and sensor data, but it also might only represent low-level operations of Apple Watch, iPhone, and study servers, such as in a regularly scheduled check-in between Research app and the servers. The presence of one of these uploads shows that the participant has Research app installed on their iPhone and that their iPhone is connected to the internet.

Figure 1 shows these two indices of participation on discrete time scales. Panel a shows the fraction of the cohort who did not participate on any given day post-enrollment (00:00:00 to 23:59:59 UTC). Panel b shows the fraction that never participates at *any time after* a given day post-enrollment and can be regarded as a measure of the incidence of cohort dropout over time, a measure of the fraction that becomes indefinitely inactive, according to that index of participation (as of the time of writing, 2022-12-01). Note that the observation window for Fig. 1 is long enough that the entire cohort has been enrolled for at least one year, but that participation after one year is not shown. The denominator in every fraction is constant: 82,809 participants.

In Fig. 1a, the fraction of the cohort whose Research app does not upload any data on day 0 is very low, around 1%, and is still relatively low (around 38%) at one-year post-enrollment. The fraction of the cohort which do not share Stand hours follows the same trend over time, but is slightly higher at all times post-enrollment, starting at 5% and increasing to 44%. The lower rate of sharing Stand hours reflects the additional requirements that the participant wears their Watch and enables sharing of Stand hours with Research app (as well as the requirement that they stand and move for 1 min that day).

Figure 1b shows a closely related trend. The fraction of the cohort whose Research app has stopped uploading any data on day 0 is <1%, increasing to 28% at one year post-enrollment. The fraction of the cohort who has indefinitely stopped sharing Stand hours on day 0 is 2%, increasing to 34% at one-year post-enrollment.

Thus, for both of these indices of participation, the decline in daily participation over time is largely attributable to the accumulation of permanently inactive participants over time, and less attributable to a degradation of study engagement among active participants. For example, 44% of the cohort does not share a Stand hour sample on day 365 post-enrollment, but this is not much larger than the 34% of the cohort that has already stopped sharing Stand hour samples at all times after day 365.

### Survey responses

Table 5 shows the percentage of participants in the cohort who complete at least one survey in their first year following enrollment, for each of the 16 survey types. Except for the 5 surveys that are triggered by rare events detected by Watch, almost all the surveys have a participation rate greater than 70%.

Figure 2 shows the response rates, vs. time since enrollment for two surveys delivered with the highest frequency—the monthly Stress Scale survey and the quarterly Changes in Health survey.

For the Stress Scale survey, only participants enrolled after 2020-05-01 are considered in order to avoid changes in delivery frequency when this initially quarterly survey became monthly after May 2020. This leads to 42,181 (the denominator for the response rate) participants. The time window we consider here extends from the individual’s enrollment date to the 12th Stress Scale survey expiration date. The scheduled delivery of this monthly survey is as follows: the first Stress Scale survey is delivered on the first Sunday of the month after enrollment and all following surveys are delivered on the first Sunday of subsequent months. Each survey expires 28 days after delivery. A decreasing trend of response rate over time is clearly visible in Fig. 2, starting at 69.55% and gradually dropping to 32.48% after one year. As expected, due to the burden of survey completion on participants, this one-year decline is larger in absolute and relative terms than the decline in active users as measured by Research app uploads in Fig. 1b, which shows only 28% of the cohort becoming indefinitely inactive at one year.

For the Changes in Health survey, the entire cohort is considered, and the time window represented by Fig. 2 extends from enrollment to 400 days later to ensure that only the first 4 quarterly surveys for each participant are counted. The quarterly surveys were distributed on the 3rd, 6th, 9th, 12th months post-enrollment. As with the Stress Scale survey, we observe a decreasing trend of response rate over in Changes in Health survey, starting at 60.69% and dropping to 34.06%.

### Changes in Health survey results

The changes in Health survey is designed to monitor various key events relevant to health, including new medical conditions, changes in medications, new injuries, and major lifestyle changes. There were 56,553 (63.8%) participants who completed at least one changes in Health survey.

Table 6 reports the number of participants indicating a change in health by three broad categories: new medical conditions, new surgical procedures, or other changes. Since the survey prompts a participant to give the date of the change, Table 6 displays different totals computed according to whether or not the participant gave a date of the change, and whether or not the date of the change coincided with their time in study. If an event is dated after enrollment, but prior to the quarterly period queried by the survey in which it is reported, then we do not exclude it from any of the counts in Table 6. Dates after the completion of the survey are coded as missing.

**Table 6:** Number of participants reporting at least one change using the Changes in Health survey by three categories of change

Reported change	Participants reporting change	Participants reporting change; missing dates removed	Participants reporting change; missing dates & pre-enrollment dates removed
Medical conditions	2168	1910	1443
Surgical Procedures	1500	1452	1294
Other Changes	51,551	48,579	44,333

After excluding events reported with no date and excluding events dated before enrollment, events in the category “Other Changes” were reported by the greatest number of people (*n* = 44,333). Events in categories “Medical Conditions” and “Surgical Procedures” were reported by fewer participants (*n* = 1443 and *n* = 1294, respectively).

Table 7 displays these events in more detail. All new medical conditions were reported with low frequency (<1% of the cohort). The most common reported new medical condition was arthritis (0.5%). New surgical procedures were also reported at low frequency. The most common new surgical procedure was “other bone surgery” (1.1%). The most commonly reported change overall was “change of insurance”, reported by 16.6% of the cohort in their first year. Additional events included in the Changes in Health survey but not shown in Table 7 were the following: a new or continued pregnancy, other medical emergencies, newly diagnosed pre-diabetes or impaired glucose tolerance, regularly smoking cigarettes, side effect of any medication or drug, or change in lower limb arthritis. Participants reporting respiratory problems were provided with follow-up survey questions to assess the duration and severity of their respiratory problem. Similarly, participants reporting a new or continued pregnancy were provided with follow-up survey questions to assess how far along they were in their pregnancy or the outcome of their pregnancy (vaginal delivery, Cesarean section, miscarriage, or other). Participants reporting a broken bone, accident, or trauma were provided with follow-up survey questions assessing which part of the body was affected or if they needed an assistive device (cane, crutches, leg braces, prosthetics, scooter, walker without wheels, wheelchair, or other assistive device).

**Table 7:** The number of participants who report a given change in health in at least one of their Changes in Health surveys

Reported change	Participants reporting change, % (*N*)
*Medical condition*	
Arthritis	0.5 (408)
Cancer	0.3 (252)
Diabetes	0.2 (194)
Neuropathy	0.2 (186)
Atrial Fibrillation	0.2 (177)
Lung Condition	0.1 (111)
Heart Attack	< 0.1 (39)
Stroke	< 0.1 (38)
Heart Failure	< 0.1 (31)
Respiratory Problems	< 0.1 (7)
*Surgical procedures*	
Other Bone, Joint or Muscle Surgery	1.1 (916)
Other Cardio Surgery	0.2 (141)
Cardiac Stent	0.1 (90)
Knee Replacement	< 0.1 (62)
Hip Replacement	< 0.1 (46)
Other Joint Replacement	< 0.1 (20)
Open Heart Surgery	< 0.1 (19)
*Other Changes*	
Change in Medical Insurance	16.6 (13,775)
Change in Employment Status or Work Responsibilities	8.5 (7048)
Change in Address	8.2 (6773)
New Primary Care or Other Doctor	7.7 (6395)
Loss of a Family Member or Other Person Close to You	7.6 (6254)
Broken Bone, Accident or Trauma	3.2 (2660)
Change in Marital Status	1.7 (1428)

Percentages are calculated with respect to the total 82,809-person cohort.

### Potential fall surveys and follow-up participation

Participants in this cohort submitted 2055 survey responses that met protocol defined criteria for follow-up before 2021-11-14, representing 1735 participants. The study received consent to follow up with 1392 surveys by phone, representing 1179 participants. Callers reached a participant in 967 of those cases, representing 829 participants (47% of the total eligible surveys, 48% of participants with eligible surveys).

### Reference demography

Recruitment patterns during this initial period resulted in some skewing of the baseline demographics which should be considered in the context of the current report. The cohort was more likely to be male (74% vs. 49% of US), white (72% vs. 60% of US), and college educated (89% of the cohort vs. 62% of US older than age 25 with > 12 years education). Ongoing recruitment continues to move the study demographics toward the national mean (data not shown) and will be described in detail in subsequent manuscripts.

Compared to traditional epidemiology or disease cohorts at the time of enrollment, the current cohort is similarly skewed, but we anticipate that with ongoing recruitment and strategies designed to correct for representation, the cohort will continue to become more representative over time. Notably, AH&MS does not enforce an upper limit on participant age and the set of participant-shared data in AH&MS is extensive. For example, the cohort has contributed ~19,300 cycling workouts, ~14,700 running workouts, ~137,000,000 heart-rate samples, and ~57,800 V_O2max_ estimates in just a single week.

### Comparison of period 2021-11-07 – 2021-11-13 to other 7-day periods

In Supplementary Fig. 2A and Supplementary Fig. 2B we report a cross-sectional description of participant data sharing for the period 2021-11-07 through 2021-11-13, and note that this period was unremarkable compared to other 7-day periods before or after it.

We performed this comparison by collecting weekly counts of 6 data types shared by the cohort between 2019-11-14 and 2022-09-01. We compared the following three passively collected HealthKit sample types: stand hours, V_O2,Max_, and mindful breathing sessions. We also looked at 3 types of workouts actively annotated: walking, yoga, and traditional strength training.

From this 6-dimensional dataset, we computed the Mahalanobis distance, *d*, of our sample week from the mean of the other 145 weeks. Assuming that each of the six variables is normally distributed, the square of this Mahalanobis distance, *d*^*2*^, should be χ^2^-distributed with ν = 6. This allows us to test for a significant difference between our sample week and the other 145 weeks. We found *d*^*2*^ = 1.57. Since $P\left({\chi }_{6}^{2} > 1.57\right)=0.954$ we find no significant difference between our week and the average study week.

We also performed a 6-component PCA on the dataset and plotted the first 2 components to graphically demonstrate the distance of our sample week from the other 145 weeks. See Supplementary Fig. 2C.

### Reporting summary

Further information on research design is available in the Nature Research Reporting Summary linked to this article.

## Supplementary information

Supplemental Material

Reporting summary

Table 1

Table 2

Table 3

Table 4

Table 5

Table 6

Table 7

Table 8

Table 9

Table 10

## Data Availability

Data are not publicly available. Any request for data will be evaluated and responded to in a manner consistent with the specific language in the study protocol and informed consent form. Requests for data should be addressed to one of the corresponding authors (CAM).

## Code availability

Computer code for all statistical analyses was written in Python and R and may be available for review upon request from one of the corresponding authors (C.A.M.). Any request for code will be evaluated and responded to in a manner consistent with policies intended to protect participant confidentiality and language in the study protocol and in the informed consent form.

## References

[1] Arnett, D. K. et al. 2019 ACC/AHA guideline on the primary prevention of cardiovascular disease: executive summary: a report of the American College of Cardiology/American Heart Association Task Force on Clinical Practice Guidelines. *J. Am. Coll. Cardiol.* **74**, 1376–1414 (2019). 30894319 10.1016/j.jacc.2019.03.009 PMC8344373

[2] Khurshid, S. et al. Wearable accelerometer-derived physical activity and incident disease. *NPJ Digit Med.* **5**, 131 (2022). 36056190 10.1038/s41746-022-00676-9 PMC9440134

[3] Master, H. et al. Association of step counts over time with the risk of chronic disease in the All of Us Research Program. *Nat. Med.* **28**, 2301–2308 (2022). 36216933 10.1038/s41591-022-02012-w PMC9671804

[4] Alemany, J. A., Delgado-Diaz, D. C., Mathews, H., Davis, J. M. & Kostek, M. C. Comparison of acute responses to isotonic or isokinetic eccentric muscle action: differential outcomes in skeletal muscle damage and implications for rehabilitation. *Int J. Sports Med.* **35**, 1–7 (2014). 23780898 10.1055/s-0032-1327652

[5] Ross, L. M., Slentz, C. A. & Kraus, W. E. Evaluating individual level responses to exercise for health outcomes in overweight or obese adults. *Front. Physiol.* **10**, 1401 (2019). 31798463 10.3389/fphys.2019.01401 PMC6867965

[6] Shigeta, T. T. et al. Cardiorespiratory and muscular fitness associations with older adolescent cognitive control. *J. Sport Health Sci.* **10**, 82–90 (2021). 32442694 10.1016/j.jshs.2020.05.004 PMC7856563

[7] Vidoni, E. D. et al. Dementia risk and dynamic response to exercise: a non-randomized clinical trial. *PLoS ONE* **17**, e0265860 (2022). 35802628 10.1371/journal.pone.0265860 PMC9269742

[8] Ross, R. et al. Precision exercise medicine: understanding exercise response variability. *Br. J. Sports Med.* **53**, 1141–1153 (2019). 30862704 10.1136/bjsports-2018-100328 PMC6818669

[9] Neufer, P. D. et al. Understanding the cellular and molecular mechanisms of physical activity-induced health benefits. *Cell Metab.* **22**, 4–11 (2015). 26073496 10.1016/j.cmet.2015.05.011

[10] Roberts, M. D. et al. Physiological differences between low versus high skeletal muscle hypertrophic responders to resistance exercise training: current perspectives and future research directions. *Front. Physiol.* **9**, 834 (2018). 30022953 10.3389/fphys.2018.00834 PMC6039846

[11] Mahalingaiah, S. et al. Design and methods of the Apple Women’s Health Study: a digital longitudinal cohort study. *Am. J. Obstet. Gynecol.* **226**, 545 e541–545.e529 (2022). 10.1016/j.ajog.2021.09.041 PMC10518829 34610322

[12] Neitzel, R. L. et al. Toward a better understanding of nonoccupational sound exposures and associated health impacts: Methods of the Apple Hearing Study. *J. Acoust. Soc. Am.* **151**, 1476 (2022). 35364926 10.1121/10.0009620

[13] Chen, T. C., Clark, J., Riddles, M. K., Mohadjer, L. K. & Fakhouri, T. H. I. National Health and Nutrition Examination Survey, 2015-2018: sample design and estimation procedures. *Vital-. Health Stat.* **2**, 1–35 (2020). 33663649

[14] All of Us Research Program, I. et al. The “All of Us” research program. *N. Engl. J. Med.* **381**, 668–676 (2019). 31412182 10.1056/NEJMsr1809937 PMC8291101

[15] Lohman, M. C. et al. Operationalisation and validation of the Stopping Elderly Accidents, Deaths, and Injuries (STEADI) fall risk algorithm in a nationally representative sample. *J. Epidemiol. Community Health* **71**, 1191–1197 (2017). 28947669 10.1136/jech-2017-209769 PMC5729578

[16] Saunders, J. B., Aasland, O. G., Babor, T. F., de la Fuente, J. R. & Grant, M. Development of the alcohol use disorders identification test (AUDIT): WHO collaborative project on early detection of persons with harmful alcohol consumption-II. *Addiction* **88**, 791–804 (1993). 8329970 10.1111/j.1360-0443.1993.tb02093.x

[17] Ware, J., Jr. Kosinski, M. & Keller, S. D. A 12-Item Short-Form Health Survey: construction of scales and preliminary tests of reliability and validity. *Med. Care* **34**, 220–233 (1996). 10.1097/00005650-199603000-00003 8628042

[18] Rosow, I. & Breslau, N. A Guttman health scale for the aged. *J. Gerontol.* **21**, 556–559 (1966). 5918309 10.1093/geronj/21.4.556

[19] Cohen, B. G., Colligan, M. J., Wester, W. 2nd & Smith, M. J. An investigation of job satisfaction factors in an incident of mass psychogenic illness at the workplace. *Occup. Health Nurs.* **26**, 10–16 (1978). 564008 10.1177/216507997802600102

[20] Andrews, G., Kemp, A., Sunderland, M., Von Korff, M. & Ustun, T. B. Normative data for the 12 item WHO Disability Assessment Schedule 2.0. *PLoS ONE* **4**, e8343 (2009). 20020047 10.1371/journal.pone.0008343 PMC2791224

[21] Adler, N. E., Epel, E. S., Castellazzo, G. & Ickovics, J. R. Relationship of subjective and objective social status with psychological and physiological functioning: preliminary data in healthy white women. *Health Psychol.* **19**, 586–592 (2000). 11129362 10.1037/0278-6133.19.6.586

