# Supplementary material for: Understanding activity and physiology at scale: The Apple Heart & Movement Study
Source: NPJ Digit Med. 2024 Sep 10;7:242. doi: 10.1038/s41746-024-01187-5 (PMC11387614; doi:10.1038/s41746-024-01187-5)
Supplement: Supplementary file 1 — Supplemental Material [file 41746_2024_1187_MOESM1_ESM.pdf]

Supplementary Figure 1

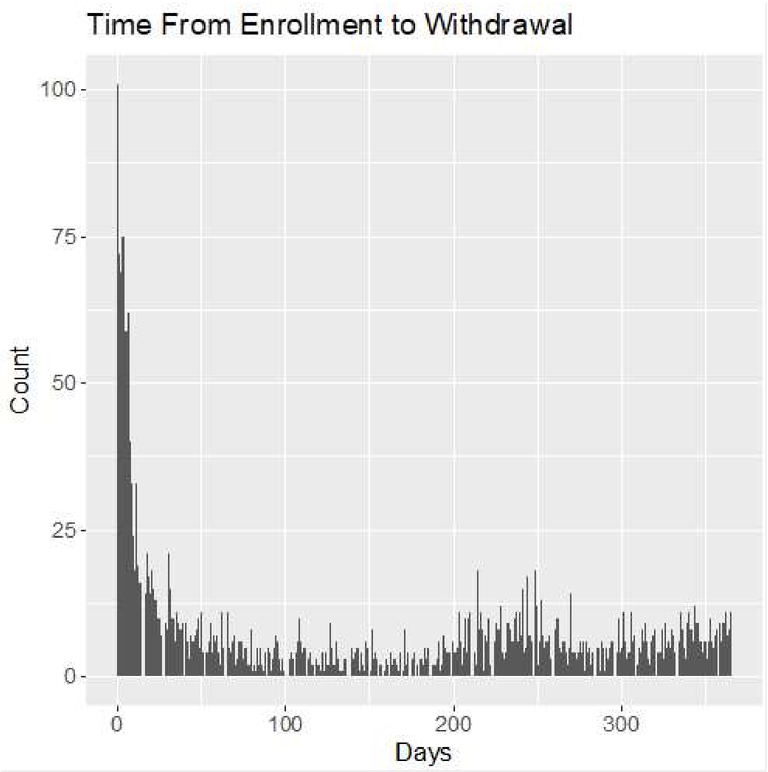

Supplementary Figure 1: Daily counts of withdrawals, vs. time since enrollment. (Withdrawals after 1 year not shown). The figure shows the number of daily withdrawals over the first year of enrollment. The plot shows a spike in withdrawals between day 0 and day 15, followed by a somewhat steady-state rate of approximately 5.8 withdrawals/day. There were 2,684 withdrawals over the first year, accounting for 3.24% of participants.

Supplementary Figure 2A

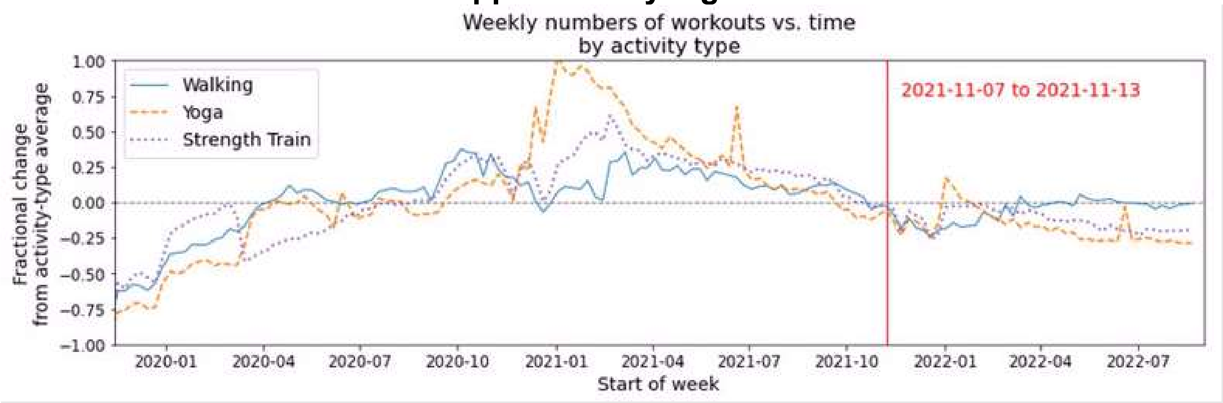

Supplementary Figure 2A: Weekly counts of 3 types of workouts. Each series is mean-centered, and then mean-scaled.

Supplementary Figure 2B

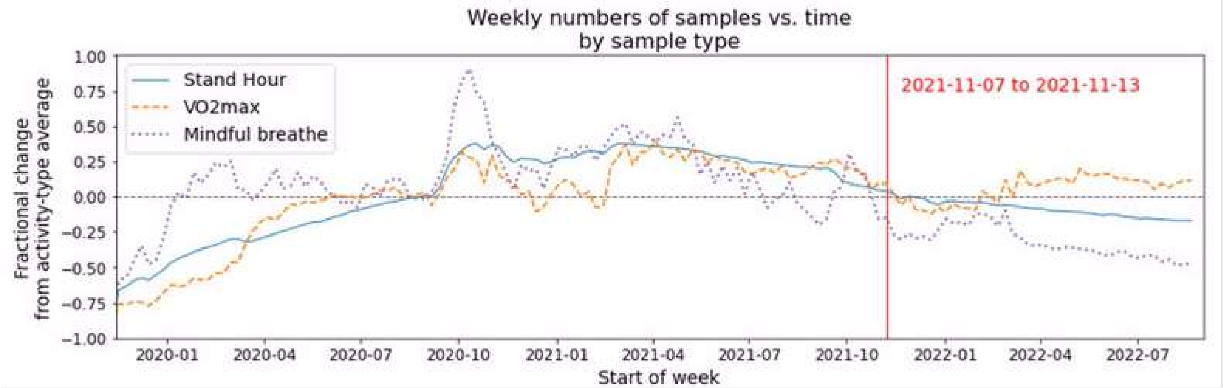

Supplementary Figure 2B: Weekly counts of 3 HealthKit data types. Each series is mean-centered, and then mean-scaled.

Supplementary Figure 2C

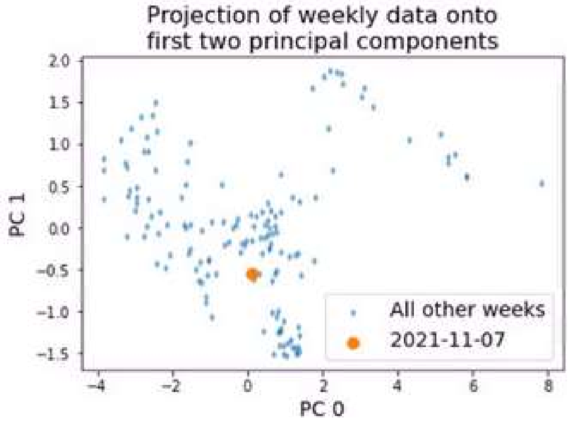

Supplementary Figure 2C: First 2 components of the 6-D PCA transform of the three workout types and three HealthKit data types.
