# Supplementary material for: Understanding activity and physiology at scale: The Apple Heart & Movement Study
Source: NPJ Digit Med. 2024 Sep 10;7:242. doi: 10.1038/s41746-024-01187-5 (PMC11387614; doi:10.1038/s41746-024-01187-5)
Supplement: Supplementary file 2 — Reporting summary [file 41746_2024_1187_MOESM2_ESM.pdf]

nature portfolio

Corresponding author(s): MacRae, CA

Last updated by author(s): May 16, 2024

Reporting Summary

Nature Portfolio wishes to improve the reproducibility of the work that we publish. This form provides structure for consistency and transparency in reporting. For further information on Nature Portfolio policies, see our Editorial Policies and the Editorial Policy Checklist.

Statistics

For all statistical analyses, confirm that the following items are present in the figure legend, table legend, main text, or Methods section.

n/a	Confirmed	
☐	☒	The exact sample size (n) for each experimental group/condition, given as a discrete number and unit of measurement
☐	☒	A statement on whether measurements were taken from distinct samples or whether the same sample was measured repeatedly
☐	☒	The statistical test(s) used AND whether they are one- or two-sided<br>*Only common tests should be described solely by name; describe more complex techniques in the Methods section.*
☐	☒	A description of all covariates tested
☐	☒	A description of any assumptions or corrections, such as tests of normality and adjustment for multiple comparisons
☐	☒	A full description of the statistical parameters including central tendency (e.g. means) or other basic estimates (e.g. regression coefficient) AND variation (e.g. standard deviation) or associated estimates of uncertainty (e.g. confidence intervals)
☐	☒	For null hypothesis testing, the test statistic (e.g. F, t, r) with confidence intervals, effect sizes, degrees of freedom and P value noted<br>*Give P values as exact values whenever suitable.*
☒	☐	For Bayesian analysis, information on the choice of priors and Markov chain Monte Carlo settings
☒	☐	For hierarchical and complex designs, identification of the appropriate level for tests and full reporting of outcomes
☐	☒	Estimates of effect sizes (e.g. Cohen's d, Pearson's r), indicating how they were calculated

Our web collection on statistics for biologists contains articles on many of the points above.

Software and code

Policy information about availability of computer code

Data collection	Not available
Data analysis	Computer code for all statistical analyses was written in Python and R and may be available for review upon request from the corresponding author (C.A.M.). Any request for code will be evaluated and responded to in a manner consistent with policies intended to protect participant confidentiality and language in the study protocol and in the informed consent form.

For manuscripts utilizing custom algorithms or software that are central to the research but not yet described in published literature, software must be made available to editors and reviewers. We strongly encourage code deposition in a community repository (e.g. GitHub). See the Nature Portfolio guidelines for submitting code & software for further information.

Data

Policy information about availability of data

All manuscripts must include a data availability statement. This statement should provide the following information, where applicable:

- Accession codes, unique identifiers, or web links for publicly available datasets
- A description of any restrictions on data availability
- For clinical datasets or third party data, please ensure that the statement adheres to our policy

Data are not publicly available. Any request for data will be evaluated and responded to in a manner consistent with the specific language in the study protocol and informed consent form.

Research involving human participants, their data, or biological material

Policy information about studies with human participants or human data. See also policy information about sex, gender (identity/presentation), and sexual orientation and race, ethnicity and racism.

Reporting on sex and gender	On enrollment, participants in AH&MS responded to the Research Profile Survey within the Research app and to the Demographics Survey; the data included the year of birth, state of residence, race and ethnicity, marital status, employment status, education level, gender identity, sex assigned at birth, and subjective social status.<br>Data on sex, gender, race and ethnicity are specified within the manuscript for each analysis performed where these are known and relevant.
Reporting on race, ethnicity, or other socially relevant groupings	We used the same questionnaire that was used to determine the race and ethnicity in the NIH-sponsored All of Us Study and we classified responses into traditional reporting of race and ethnicity. All three of the Research app studies used the MacArthur Scale of Subjective Social Status. This scale has been observed to correlate with health status across the lifespan. Notably, the MacArthur Scale is correlated with objective socioeconomic status (SES) but has the benefit of broader applicability as a marker of social status than simple objective measures of SES in non-White populations. We arbitrarily categorized the responses into the following categories: 1 to 4 corresponding to low, 5 to 6 corresponding to middle, and 7 to 10 corresponding to high.
Population characteristics	Eligibility criteria include access to an iPhone with Research app installed, comfort communicating in written and spoken English, residence in the United States, aged at least 18 years old (at least 19 years old in Alabama and Nebraska, at least 21 years old in Puerto Rico), the sole users of their iCloud account or iPhone, and willing to provide informed consent to participate in the study. An additional requirement for AH&MS includes use of an Apple Watch (Series 1 or later) paired with an iPhone at the time of enrollment.<br>Prior to enrollment, participants complete a profile which includes information such as name, date of birth, email, phone number, current region and state of residence. These data are used to confirm eligibility. For AH&MS, Research app is also able to confirm if an Apple Watch is paired to the iPhone. If the requirements for age, location, and Watch pairing status are met, individuals are able to continue to study onboarding, including reading and signing the informed consent form (ICF), HIPAA Authorization, and California Bill of Rights (if applicable).
Recruitment	Similar to the Apple Women's Health Study and Apple Hearing Study eligibility criteria include access to an iPhone with Research app installed, comfort communicating in written and spoken English, residence in the United States, aged at least 18 years old (at least 19 years old in Alabama and Nebraska, at least 21 years old in Puerto Rico), the sole users of their iCloud account or iPhone, and willing to provide informed consent to participate in the study. An additional requirement for AH&MS includes use of an Apple Watch (Series 1 or later) paired with an iPhone at the time of enrollment.
Ethics oversight	Advarra Central Institutional Review Board (PRO00036784)

Note that full information on the approval of the study protocol must also be provided in the manuscript.

Field-specific reporting

Please select the one below that is the best fit for your research. If you are not sure, read the appropriate sections before making your selection.

☒ Life sciences ☐ Behavioural & social sciences ☐ Ecological, evolutionary & environmental sciences

For a reference copy of the document with all sections, see nature.com/documents/nr-reporting-summary-flat.pdf

Life sciences study design

All studies must disclose on these points even when the disclosure is negative.

Sample size	Varies with the specific analysis but defined in each case.
Data exclusions	Only exclusions made were those specified within the text for the relevant analyses.
Replication	All findings were replicated by multiple team members. We did not have access to an independent data set but we did split the datasets into tiered derivation and validation cohorts where appropriate based on cohort selection.
Randomization	Not applicable
Blinding	Observational study-blinding post hoc not relevant.

Reporting for specific materials, systems and methods

We require information from authors about some types of materials, experimental systems and methods used in many studies. Here, indicate whether each material, system or method listed is relevant to your study. If you are not sure if a list item applies to your research, read the appropriate section before selecting a response.

Materials & experimental systems

n/a	Involved in the study
☒	☐ Antibodies
☒	☐ Eukaryotic cell lines
☒	☐ Palaeontology and archaeology
☒	☐ Animals and other organisms
☐	☒ Clinical data
☒	☐ Dual use research of concern
☒	☐ Plants

Methods

n/a	Involved in the study
☒	☐ ChIP-seq
☒	☐ Flow cytometry
☒	☐ MRI-based neuroimaging

Clinical data

Policy information about clinical studies

All manuscripts should comply with the ICMJE guidelines for publication of clinical research and a completed CONSORT checklist must be included with all submissions.

Clinical trial registration: ClinicalTrials.gov Identifier: NCT04198194

Study protocol: See current manuscript for details

Data collection: The Apple Health app allows users to download clinical health records (FHIR format) from participating institutions by signing into their healthcare provider's portal and choosing to share FHIR data with HealthKit. Study participants may elect to share this data with our study. To date, the proportion of participants who have been able to share these data types is modest (~10%) as a consequence of local FHIR compliance and the process required. In the cohort, 7,757 people shared at least one such record with our study in their initial year post-enrollment.

Outcomes: We do not describes any outcomes in the current manuscript.

Plants

Seed stocks: *Report on the source of all seed stocks or other plant material used. If applicable, state the seed stock centre and catalogue number. If plant specimens were collected from the field, describe the collection location, date and sampling procedures.*

Novel plant genotypes: *Describe the methods by which all novel plant genotypes were produced. This includes those generated by transgenic approaches, gene editing, chemical/radiation-based mutagenesis and hybridization. For transgenic lines, describe the transformation method, the number of independent lines analyzed and the generation upon which experiments were performed. For gene-edited lines, describe the editor used, the endogenous sequence targeted for editing, the targeting guide RNA sequence (if applicable) and how the editor was applied.*

Authentication: *Describe any authentication procedures for each seed stock used or novel genotype generated. Describe any experiments used to assess the effect of a mutation and, where applicable, how potential secondary effects (e.g. second site T-DNA insertions, mosiacism, off-target gene editing) were examined.*
