# Supplementary material for: Understanding activity and physiology at scale: The Apple Heart & Movement Study
Source: NPJ Digit Med. 2024 Sep 10;7:242. doi: 10.1038/s41746-024-01187-5 (PMC11387614; doi:10.1038/s41746-024-01187-5)
Supplement: Supplementary file 3 — Table 1 [file 41746_2024_1187_MOESM3_ESM.docx]

**Supplementary Table 1**

| **Mode of Participant Data Collection** | **At enrollment** | **Within first year** | **Annually** | **Quarterly** | **Daily** | **Qualifying Event** | **Every 2 years** | **Qualifying IRB Amendment** |
| --- | --- | --- | --- | --- | --- | --- | --- | --- |
| Informed Consent | X |  |  |  |  |  | X | X |
| Onboarding - Getting Started | X |  |  |  |  |  |  |  |
| Request for Passive Data Collection - Priority | X |  |  |  |  |  |  |  |
| Request for Passive Data Collection - Lower Priority |  | X |  |  |  |  |  |  |
| Passive Data Collection |  |  |  |  | X |  |  |  |
| Research Profile (age, location) | X |  |  |  |  |  |  |  |
| Demographics survey | X |  | X |  |  |  |  |  |
| Risk of Falling survey |  | X | X |  |  |  |  |  |
| Medical History survey |  | X | X |  |  |  |  |  |
| Medications survey |  | X | X |  |  |  |  |  |
| Health Behaviors survey |  | X | X |  |  |  |  |  |
| Physical Activity survey |  | X | X |  |  |  |  |  |
| Mental Health survey |  |  |  | X |  |  |  |  |
| Activity Status survey |  |  |  | X |  |  |  |  |
| Stress Scale survey |  |  |  | X |  |  |  |  |
| Disability Assessment survey |  |  |  | X |  |  |  |  |
| Changes in Health survey |  |  |  | X |  |  |  |  |
| Triggered: Potential Fall survey |  |  |  |  |  | X |  |  |
| Triggered: Irregular Rhythm Follow-up survey |  |  |  |  |  | X |  |  |
| Triggered: Take an ECG survey |  |  |  |  |  | X |  |  |
| Triggered: ECG Follow-up survey |  |  |  |  |  | X |  |  |

**Supplementary Table 1:** Various modes of data collection, and conditions under which they occur. The Physical Activity survey changed from annual to quarterly in October 2020. The Stress Scale survey changed from quarterly to monthly in May 2020.
