# Supplementary material for: Understanding activity and physiology at scale: The Apple Heart & Movement Study
Source: NPJ Digit Med. 2024 Sep 10;7:242. doi: 10.1038/s41746-024-01187-5 (PMC11387614; doi:10.1038/s41746-024-01187-5)
Supplement: Supplementary file 4 — Table 2 [file 41746_2024_1187_MOESM4_ESM.docx]

**Supplementary Table 2**

| Data type | What is collected | What is not collected |
| --- | --- | --- |
| Mandatory | | |
| Distance and Speed | •Speed, distance, altitude, slope of motion.  •When your motion changes and whether you’re walking or running.  •The type of device you’re using and when the data was collected.  •Altitude and accuracy information related to GPS sensor. | •Specific location information such as places visited or latitude and longitude. |
| ECG Details | •The data of your ECG recording.  •Your ECG results. | •Any additional heart information. |
| Elevation | •Your barometric pressure and change in elevation over a period of time. | •Specific location information such as places visited or latitude and longitude. |
| Motion | •High-fidelity rotation rate and accelerometry data from your iPhone and Apple Watch | •GPS or specific location information. |
| Optical sensor | •High-fidelity Apple Watch optical sensor data that can be used to determine things like heart rate and blood oxygen saturation  •High-fidelity accelerometer data  •Calculated heart rate, along with reading accuracy | •ECG data or results. |
| Secondary | | |
| Fall Detection Details | •The characteristics of your fall and your response to the alert on your watch  •High-fidelity rotation rate and accelerometer data as well as air pressure and whether or not you’re wearing your Apple Watch and its orientation  •Your heart rate around the time of your fall | •How you use your Apple Watch. |
| Frequently Visited Locations | •Frequently visited locations but only after they are given an anonymized identifier.  •The distance of an anonymized location from your home.  •Arrival and departure times that are expanded to 15-min increments to make them less specific. | •GPS or specific location information.  •Names of the places you’ve visited.  •Addresses including home or work. |
| Pedometer | •Your step count | •GPS or specific location information. |
| Watch on Wrist | •When you wear your Apple Watch as well as your wrist and Digital Crown settings | •How you use your Apple Watch |

**Supplementary Table 2**: Sensor-based data types collected by Apple Heart and Movement Study at study launch as of November 2019.
