# Supplementary material for: Understanding activity and physiology at scale: The Apple Heart & Movement Study
Source: NPJ Digit Med. 2024 Sep 10;7:242. doi: 10.1038/s41746-024-01187-5 (PMC11387614; doi:10.1038/s41746-024-01187-5)
Supplement: Supplementary file 5 — Table 3 [file 41746_2024_1187_MOESM5_ESM.docx]

**Supplementary Table 3**

|  | **Excluded** | **Included** |
| --- | --- | --- |
| **Total** | 1,751 | 82,809 |
| **Age (yrs),** Mean ± SD | 36.1 ± 12.2 | 39.3 **±** 13.1 |
| **Region,** % *(N)* |  |  |
| Midwest | 15.6 (273) | 18.7 (15,487) |
| Northeast | 16.6 (291) | 17.2 (14,222) |
| South | 35.1 (615) | 34.6 (28,653) |
| Territories | 0.5 (10) | 1.9 (158) |
| West | 31.9 (559) | 29.3 (24,280) |
| Missing | 0.2 (3) | 0.1 (9) |

**Supplementary Table 3**: Age and place of residence at enrollment. The 82,809-person cohort is compared to the 1,751 candidates who met all inclusion criteria except for responding to a Demographics survey. In this table, the label “excluded” denotes the 1,751-person group, and the label “included” denotes the 82,809-person cohort.
