# Supplementary material for: Understanding activity and physiology at scale: The Apple Heart & Movement Study
Source: NPJ Digit Med. 2024 Sep 10;7:242. doi: 10.1038/s41746-024-01187-5 (PMC11387614; doi:10.1038/s41746-024-01187-5)
Supplement: Supplementary file 6 — Table 4 [file 41746_2024_1187_MOESM6_ESM.docx]

**Supplementary Table 4**

| ***t*** | **DoF** | ***p*-value** | **95% CI** | **N_excluded_** | **N_included_** |
| --- | --- | --- | --- | --- | --- |
| -10.7 | 1836.3 | <0.0001 | (-3.74, -2.58) | 1,715 | 82,809 |

**Supplementary Table 4:** Reports results from Welch two-sample t-test comparing mean age at enrollment between excluded and included groups in Supplementary Table 4. The mean age of excluded participants is between 3.79 and 2.58 years younger than included participants (p-value < 0.0001).
