# Supplementary material for: Understanding activity and physiology at scale: The Apple Heart & Movement Study
Source: NPJ Digit Med. 2024 Sep 10;7:242. doi: 10.1038/s41746-024-01187-5 (PMC11387614; doi:10.1038/s41746-024-01187-5)
Supplement: Supplementary file 7 — Table 5 [file 41746_2024_1187_MOESM7_ESM.docx]

**Supplementary Table 5**

| **χ^2^** | **DoF** | ***p*-value** | **N_excluded_** | **N_included_** |
| --- | --- | --- | --- | --- |
| 59.123 | NA | <0.0001 | 1,751 | 82,809 |

**Supplementary Table 5**: Reports results from Pearson’s chi-squared test with simulated p-values comparing the distribution of geographic regions at enrollment among excluded and included groups of participants in Supplementary Table 4. The chi-squared test yielded a test statistic of 59.1, indicating a statistically significant difference in geographic locations between included and excluded participants.
