# Supplementary material for: Understanding activity and physiology at scale: The Apple Heart & Movement Study
Source: NPJ Digit Med. 2024 Sep 10;7:242. doi: 10.1038/s41746-024-01187-5 (PMC11387614; doi:10.1038/s41746-024-01187-5)
Supplement: Supplementary file 8 — Table 6 [file 41746_2024_1187_MOESM8_ESM.docx]

**Supplementary Table 6**

| **Category** | **Participants, % (*N*)** |
| --- | --- |
| not Hispanic | 88.7 (73,463) |
| Hispanic only | 7.7 (6,344) |
| Hispanic-White | 2.5 (2,091) |
| Hispanic-Mixed | 0.6 (489) |
| Hispanic-Black | 0.2 (201) |
| Hispanic-other | 0.2 (143) |
| Hispanic-Asian | < 0.1 (78) |

**Supplementary Table 6:** Frequency with which participants self-identified as Hispanic, along with any other category with which they self-identified.
