# Supplementary material for: Understanding activity and physiology at scale: The Apple Heart & Movement Study
Source: NPJ Digit Med. 2024 Sep 10;7:242. doi: 10.1038/s41746-024-01187-5 (PMC11387614; doi:10.1038/s41746-024-01187-5)
Supplement: Supplementary file 9 — Table 7 [file 41746_2024_1187_MOESM9_ESM.docx]

**Supplementary Table 7**

| **Survey Response** | **Prevalence, % (N)** |
| --- | --- |
| Any medication | 46.8 (38,743) |
| NSAIDs | 20.6 (17,088) |
| Antidepressants | 15.5 (12,875) |
| ACE-inhibitors or ARBs | 8.6 (7,120) |
| Antianxiety | 7.8 (6,431) |
| Beta-blockers | 5.8 (4,835) |
| Diuretics | 4.6 (3,840) |
| Antiplatelets | 4.5 (3,712) |
| Sleep medications | 4.4 (3,620) |
| Ca+ channel blocker | 3.1 (2,564) |
| Painkiller injections | 2.4 (1,998) |
| Other blood-pressure meds | 2.0 (1,630) |
| Opioids | 1.9 (1,589) |
| Antipsychotic | 1.7 (1,386) |
| Anticoagulants | 1.5 (1,250) |
| Chemotherapy | 0.3 (231) |
| No survey submitted | 23.6 (19,534) |

**Supplementary Table 7**: Current medication use, as reported in the Medications survey.
