# Supplementary material for: Understanding activity and physiology at scale: The Apple Heart & Movement Study
Source: NPJ Digit Med. 2024 Sep 10;7:242. doi: 10.1038/s41746-024-01187-5 (PMC11387614; doi:10.1038/s41746-024-01187-5)
Supplement: Supplementary file 10 — Table 8 [file 41746_2024_1187_MOESM10_ESM.docx]

**Supplementary Table 8**

| **Sample Type** | **Subjects,  % (N)** | **Samples/ Subject** | **Sample Type** | **Subjects,  % (N)** | **Samples/ Subject** |
| --- | --- | --- | --- | --- | --- |
| Step count | 54.2 (44,843) | 997.1 | Dietary energy consumed | 3.6 (2,967) | 25.1 |
| Distance walking running | 53.9 (44,665) | 2,177.8 | Dietary carbohydrates | 3.3 (2,696) | 22.9 |
| Active energy burned | 51.6 (42,754) | 7,981.2 | Dietary protein | 3.2 (2,652) | 22.8 |
| Heart rate | 51.5 (42,613) | 3,219.1 | Dietary sugar | 3.2 (2,633) | 21.6 |
| Basal energy burned | 51.4 (42,588) | 4,208.5 | Dietary fat total | 3.2 (2,630) | 22.7 |
| Flights climbed | 51.4 (42,544) | 52.1 | Dietary fiber | 3.2 (2,629) | 21.4 |
| Apple stand hour | 51.1 (42,321) | 114.8 | Dietary sodium | 3.2 (2,624) | 22.3 |
| Apple stand time | 50.9 (42,180) | 336.8 | Dietary cholesterol | 3.1 (2,592) | 20.6 |
| Apple exercise time | 50.9 (42,180) | 237.7 | Dietary fat saturated | 3.1 (2,573) | 21.8 |
| Heart rate variability SDNN | 50.8 (42,092) | 36.3 | High heart rate event | 2.4 (2,002) | 4.3 |
| Resting heart rate | 50.7 (42,018) | 6.6 | Blood pressure diastolic | 2.4 (1,985) | 12.1 |
| Walking heart rate average | 50.5 (41,796) | 6.3 | Blood pressure systolic | 2.4 (1,976) | 12.1 |
| Walking speed | 49.1 (40,698) | 168.5 | Dietary water | 2.4 (1,961) | 33.4 |
| Walking step length | 49.1 (40,662) | 168.5 | Dietary fat polyunsaturated | 2.0 (1,666) | 14.4 |
| Walking double support percentage | 48.9 (40,490) | 118.0 | Dietary fat monounsaturated | 2.0 (1,666) | 14.4 |
| Walking asymmetry percentage | 47.9 (39,657) | 50.0 | Menstrual flow | 1.9 (1,580) | 3.9 |
| Six-minute walk test distance | 37.1 (30,701) | 1.0 | Height | 1.6 (1,302) | 4.9 |
| Sleep analysis | 36.8 (30,471) | 40.6 | Low heart rate event | 1.2 (960) | 15.0 |
| Stair-ascent speed | 30.5 (25,241) | 26.3 | Blood glucose | 1.1 (895) | 624.7 |
| Stair-descent speed | 30.2 (25,002) | 26.1 | Sexual activity | 0.9 (749) | 2.0 |
| Respiratory rate | 26.6 (22,017) | 162.3 | Dietary caffeine | 0.7 (618) | 9.4 |
| VO2 max | 22.2 (18,350) | 3.3 | Body temperature | 0.6 (527) | 5.2 |
| Oxygen saturation | 21.5 (17,792) | 57.8 | Distance swimming | 0.6 (493) | 120.9 |
| Hand washing event | 20.4 (16,882) | 17.8 | Swimming stroke count | 0.6 (487) | 123.8 |
| Body mass | 11.5 (9,560) | 4.6 | Intermenstrual bleeding | 0.4 (348) | 1.8 |
| Body mass index | 8.1 (6,692) | 4.5 | Sleep duration goal | 0.2 (156) | 1.0 |
| Body fat percentage | 6.4 (5,277) | 4.0 | Waist circumference | 0.2 (130) | 1.8 |
| Mindful session | 5.5 (4,523) | 6.6 | Number of times fallen | 0.2 (127) | 1.2 |
| Lean body mass | 5.3 (4,416) | 3.8 | Irregular heart rhythm event | 0.1 (108) | 8.3 |
| Distance cycling | 4.2 (3,439) | 3,238.4 |  |  |  |

**Supplementary Table 8:** 59 types of data shared by at least 100 participants during final 7 days of observation period, 2021-11-07 through 2021-11-13. *Variable name*: the label used within HealthKit for a given variable. *Subjects*: How many people, *N,* shared at least one such sample (i.e. at least one measurement of that variable). And *N* as a percentage of 82,809-person cohort. *Samples per subject*: Total number of measurements divided by *N.*
