# Supplementary material for: Understanding activity and physiology at scale: The Apple Heart & Movement Study
Source: NPJ Digit Med. 2024 Sep 10;7:242. doi: 10.1038/s41746-024-01187-5 (PMC11387614; doi:10.1038/s41746-024-01187-5)
Supplement: Supplementary file 11 — Table 9 [file 41746_2024_1187_MOESM11_ESM.docx]

**Supplementary Table 9**

| **Label** | **Number of ECGs with each label, by participant’s age group, *N* (%)** | | | | | | |
| --- | --- | --- | --- | --- | --- | --- | --- |
|  | All Ages | 18-24 | 25-34 | 35-44 | 45-54 | 55-64 | 65+ |
| All Labels | 1,132,473 (100.0) | 100,974 (100.0) | 267,700 (100.0) | 285,043 (100.0) | 204,669 (100.0) | 142,382 (100.0) | 131,705 (100.0) |
| Sinus Rhythm | 925,634 (81.7) | 83,347 (82.5) | 223,969 (83.7) | 241,426 (84.7) | 170,605 (83.4) | 111,529 (78.3) | 94,758 (71.9) |
| Inconclusive High Heart Rate | 15,566 (1.4) | 1,815 (1.8) | 3,973 (1.5) | 3,432 (1.2) | 2,544 (1.2) | 2,221 (1.6) | 1,581 (1.2) |
| Inconclusive Low Heart Rate | 22,426 (2.0) | 1,175 (1.2) | 4,762 (1.8) | 4,219 (1.5) | 4,516 (2.2) | 3,666 (2.6) | 4,088 (3.1) |
| Atrial Fibrillation | 25,402 (2.2) | 530 (0.5) | 975 (0.4) | 1,403 (0.5) | 3,192 (1.6) | 6,334 (4.4) | 12,968 (9.8) |
| Inconclusive Poor Reading | 60,038 (5.3) | 5,458 (5.4) | 13,839 (5.2) | 13,686 (4.8) | 10,570 (5.2) | 8,394 (5.9) | 8,091 (6.1) |
| Inconclusive Other | 83,407 (7.4) | 8,649 (8.6) | 20,182 (7.5) | 20,877 (7.3) | 13,242 (6.5) | 10,238 (7.2) | 10,219 (7.8) |

**Supplementary Table 9**: The 1,132,473 ECGs recorded by the cohort within the first year post-enrollment. The label given to each ECG by Watch software is given in the leftmost column. (Each ECG is assigned exactly one label.) The age of the participant with that ECG is given in the top row. Denominator for each percentage is the total count for the age group, across all labels.
