# Supplementary material for: Understanding activity and physiology at scale: The Apple Heart & Movement Study
Source: NPJ Digit Med. 2024 Sep 10;7:242. doi: 10.1038/s41746-024-01187-5 (PMC11387614; doi:10.1038/s41746-024-01187-5)
Supplement: Supplementary file 12 — Table 10 [file 41746_2024_1187_MOESM12_ESM.docx]

**Supplementary Table 10**

| **Label** | **Number of unique participants with each ECG label, by age group** | | | | | | |
| --- | --- | --- | --- | --- | --- | --- | --- |
|  | All Ages | 18-24 | 25-34 | 35-44 | 45-54 | 55-64 | 65+ |
| Sinus Rhythm | 54,795 | 6,129 | 16,401 | 15,250 | 9,436 | 4,558 | 3,021 |
| Inconclusive High Heart Rate | 5,247 | 748 | 1,709 | 1,383 | 773 | 384 | 250 |
| Inconclusive Low Heart Rate | 4,613 | 365 | 1,137 | 1,173 | 834 | 561 | 543 |
| Atrial Fibrillation | 1,641 | 136 | 258 | 267 | 262 | 291 | 427 |
| Inconclusive Poor Reading | 18,230 | 2,058 | 4,959 | 4,707 | 3,184 | 1,853 | 1,469 |
| Inconclusive Other | 16,884 | 2,116 | 4,896 | 4,569 | 2,729 | 1,422 | 1,152 |

**Supplementary Table 10**: Derived from the same dataset as used in Supplementary Table 9. However here, for each label, the table shows the number of participants who record at least one ECG with that label. In general, a participant can appear on multiple rows in this table, depending on how many types of ECG they record.
